# Modular Set of Reagents in Lateral Flow Immunoassay: Application for Antibiotic Neomycin Detection in Honey

**DOI:** 10.3390/bios13050498

**Published:** 2023-04-25

**Authors:** Lyubov V. Barshevskaya, Dmitriy V. Sotnikov, Anatoly V. Zherdev, Boris B. Dzantiev

**Affiliations:** A.N. Bach Institute of Biochemistry, Research Center of Biotechnology of the Russian Academy of Sciences, Leninsky Prospect 33, 119071 Moscow, Russia; lyubov.barshevskaya@yandex.ru (L.V.B.); zherdev@inbi.ras.ru (A.V.Z.)

**Keywords:** lateral flow assay, neomycin, antibiotics, honey, streptavidin, anti-species antibodies

## Abstract

A scheme of modular competitive immunochromatography with an analyte-independent test strip and changeable specific immunoreactants has been proposed. Native (detected) and biotinylated antigens interact with specific antibodies during their preincubation in solution, that is, without the immobilization of reagents. After this, the detectable complexes on the test strip are formed by the use of streptavidin (which binds biotin with high affinity), anti-species antibodies, and immunoglobulin-binding streptococcal protein G. The technique was successfully applied for the detection of neomycin in honey. The visual and instrumental detection limits were 0.3 and 0.014 mg/kg, respectively, and the degree of neomycin revealed in honey samples varied from 85% to 113%. The efficiency of the modular technique with the use of the same test strip for different analytes was confirmed for streptomycin detection. The proposed approach excludes the necessity of finding the condition of immobilization for each new specific immunoreactant and transferring the assay to other analytes by a simple choice of concentrations for preincubated specific antibodies and the hapten–biotin conjugate.

## 1. Introduction

The lateral flow immunoassay (immunochromatographic assay) is an analytical approach in high demand [1,2]. Immunochromatographic test systems use reagents that are preapplied to the components of a membrane test strip or are immobilized on marker nanoparticles. Because of this, mixing the characterized sample with a single diluting solution, and contact between the test strip and this mixture, initiates the movement of the liquid along the membranes, all the necessary interactions, and the formation of a detectable complex as a colored zone of the test strip. This principle minimizes the labor intensity of the assays and allows the rapid obtaining of their results [3]. The advantages of immunochromatography allow expanding applications for primary screening control. Test systems for the detection of a wide variety of compounds, from choriogonadotropin (a pregnancy biomarker) to antibodies against SARS-CoV-2, are successfully used in mass practice. Leading manufacturers offer dozens and hundreds of test systems of different selectivity [4,5]. However, developments of test systems for new analytes, that is, with new reagents, are accompanied by difficulties. An integral part of the reagents’ preparation for immunochromatography is the immobilization of reagents on membrane carriers and marker nanoparticles. Although standard techniques for these processes are described [6,7], they often need to be adapted to the properties of specific molecules by changing the pH of the reaction medium, its composition, and its ionic strength, as well as through the choice of reagents concentrations. Without such adaptation, a significant part of the immobilized molecules can be inactivated, or colloid preparations can aggregate [8]. For example, references [9,10] described that only 17–34% and 23% of antibodies retain their antigen-binding ability for widely used adsorptive immobilization of antibodies on gold nanoparticles (GNPs), respectively, the most common immunochromatographic marker. Some kinds of biosensors realize immobilization-free approaches (see [11,12,13] as examples). However, in the case of LFIA, to create detectable complexes in the analytical zone, we should use direct (before assay) or indirect (before assay or in the course of the assay) binding of immunoreactants with membrane surface and nanoparticle surface. Therefore, the problem of immobilization-caused inactivation is only shifted to other molecules but cannot be excluded for LFIA.

In this regard, variants of immunochromatographic test systems are in demand, in which only universal (independent of the determined compound) reagents are immobilized. In this way, it becomes possible after choosing the immobilization conditions for a small number of these reagents to use the same test strip in different assays by its combination with solutions of native antigen-specific reagents. This approach excludes the necessity to adapt the immobilization protocols for new reagents. The idea listed above was successfully realized in immunochromatographic tests that detect specific antibodies (immunoglobulins E) to allergens [14]. The test kits for controlling allergies to various compounds contain biotinylated antigens, a conjugate marker nanoparticle, and anti-IgE antibodies. The test strip does not contain specific allergens, and the formation of a detectable colored zone is provided by immobilized streptavidin. This approach is successfully used in test systems manufactured by Dr. Fooke’s company [15].

To fill this gap, we propose a universal modular set of reagents for the immunochromatography of low-molecular-weight compounds. Because of the immunochemical monovalence of such substances, they are detected in a competitive format when the antigen in the sample and the antigen derivative conjugated with some protein carrier compete for binding with specific antibodies. When the test strip for common competitive immunochromatography is prepared, the antigen–protein conjugate is immobilized in the analytical (test) zone, and specific antibodies are immobilized on nanoparticle labels [16]. However, proteins often lose their reactivity after immobilization due to conformational rearrangements or the inaccessibility of binding sites for interactions [8,9,10]. Therefore, for each new immunochromatographic test system, the immobilization of used proteins requires a specific study and selection of optimal conditions.

**Figure 1:**
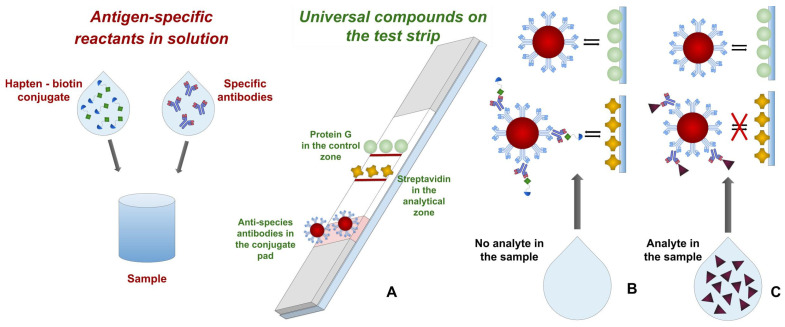
Scheme of universal competitive immunochromatography: (**A**)—compounds used; (**B**)—assay for samples without analyte or with analyte content lower than threshold level, (**C**)—assay for samples with analyte content higher than threshold level. The solutions of specific antibodies and hapten–biotin conjugate are added to the sample with analyte, after that the test strip is placed into the mixture. The reagents move with the liquid along the test strip, elute conjugate of GNPs with anti-species antibodies, and form GNP-labeled complexes with streptavidin and protein G immobilized in analytical and control zones, respectively.

We propose carrying out interactions of native (detected) and biotinylated antigens with specific antibodies during their preincubation in solution, that is, without the immobilization of reagents. After this, the detectable complexes on the test strip are formed by the use of streptavidin (which binds biotin with high affinity), anti-species antibodies, and immunoglobulin-binding streptococcal protein G. The scheme of the proposed modular test system is given in Figure 1, and interactions in it are commented on in detail at the beginning of the Results and Discussion section.

In this work, the development of a modular immunochromatographic test system and the assessment of its application for real samples were carried out for the task of honey control. The availability of rapid tests for beekeeping has become extremely important in recent years, characterized by the mass death of bees due to widespread infections and environmental contamination [17,18]. Moreover, confirmation of safety for honey is in high demand due to its decentralized production [19]. Neomycin, an aminoglycoside antibiotic with multiple toxic effects widely used in beekeeping [20,21], was chosen as the target antigen.

The presented study included obtaining and characterizing reactants for the proposed modular immunochromatographic test system, studies of their interaction and the choice of the optimal conditions of the assay, and final testing of honey samples that demonstrated the efficiency of the developed assay.

## 2. Materials and Methods

### 2.1. Reagents and Materials

The following reagents were used: Chloroauric acid, neomycin trisulfate (NEO), Tween-20, biotinamidohexanoyl-6-aminohexanoic acid *N*-hydroxysuccinimide ester, sodium citrate (Sigma-Aldrich, St. Louis, MO, USA), streptomycin sulfate (AppliChem, Darmstadt, Germany), bovine serum albumin (BSA) (Boval Biosolutions, Cleburne, TX, USA), streptavidin (Arista Biologicals, Allentown, PA, USA), streptococcal protein G, goat anti-mouse antibodies (GAMI), and anti-mouse antibodies labeled with horseradish peroxidase (Imtek, Moscow, Russia). Anti-NEO monoclonal antibodies and the NEO–BSA conjugate, monoclonal antibodies to streptomycin, and the streptomycin–BSA conjugate were from Eximio Biotec (Wuxi, China). Salts, acids, and alkalis were of analytical or chemical grade. Solutions of GNPs and their conjugates were prepared using deionized water purified by the Simplicity system (Millipore, Billerica, MA, USA). The set of immunochromatographic membranes included the UniSart CN180 working nitrocellulose membrane (Sartorius, Goettingen, Germany), the GFB-R7L sample membrane, the AP-045 adsorption membrane, and the L-P25 plastic support (Advanced Microdevices, Ambala Cantt, India).

### 2.2. Syntheses of Gold Nanoparticles

GNPs were obtained according to the Frens method [22]. First, 100 mL of a 0.01% HAuCl4 solution was heated to boiling, and then 1.5 mL of a 1% sodium citrate solution was added with vigorous stirring. The mixture was boiled for 15 min and then cooled and stored at 4 °C.

### 2.3. Conjugation of Gold Nanoparticles with Goat Anti-Mouse Antibodies

The GNP solution was adjusted to pH 8.5–9.0. GAMI was dialyzed against a 10 mM Tris-HCl buffer, pH 8.0, and added (final concentration of 10 μg/mL) to GNPs. The mixture was incubated for 45 min, and then a 10% BSA solution was added and kept under vigorous stirring for another 10 min. GNPs were precipitated by centrifugation at 13,400× *g* and 4 °C for 15 min. The pellets were collected, and then twice resuspended in 10 mM Tris buffer, pH 8.5, with 1% BSA and 1% sucrose (TBSA) and centrifuged again. The final precipitate was resuspended in TBSA with 0.05% sodium azide and stored at 4 °C.

### 2.4. Transmission Electron Microscopy

Images of GNPs were obtained using an electron microscope CX-100 (Jeol, Tokyo, Japan) at an accelerating voltage of 80 kV and a magnification of 33,000. The photographs were scanned and analyzed using the Image Tool program (UTHSCSA, San Antonio, TX, USA). The obtained results are shown in the Supplementary Material (Figure S1).

### 2.5. Synthesis of Neomycin-Biotin Conjugate

Neomycin trisulfate (contains 6 amino groups per 1 molecule) was taken in a quantity of 0.6 mg (0.66 µM), and biotinamidohexanoyl-6-aminohexanoic acid *N*-hydroxysuccinimide ester was taken in a quantity of 3 mg (5 µM). Then both preparations were added to 50 µL of C_2_H_5_OH and incubated for 1 h. The activated biotin derivative was added in excess to minimize the number of unreacted neomycin amino groups. Next, 200 µL of the 10% BSA solution (0.3 µM) was added to the reaction mixture and incubated for 30 min. One BSA molecule contains more than 30 surface amino groups [23]. That is, 0.3 µM BSA is able to bind more than 9 µM of activated biotin. The resulting conjugate was purified using Amicon Ultracel 10 K filters (Millipore, USA), centrifuging the reaction mixture at 10,000× *g* for 15 min. The solution passed through the filter containing the target neomycin–biotin conjugate, whereas BSA, which has bound excess biotin, remained on the filter. The same methodology was used to synthesize streptomycin-biotin conjugate.

### 2.6. Preparation of Immunochromatographic Test Strips

An analytical zone was formed on a working nitrocellulose membrane using an IsoFlow automatic dispenser (Imagene Technology, Lebanon, NH, USA) and a streptavidin solution (1 mg/mL, application volume 1 μL/mm) in a 50 mM potassium phosphate buffer, pH 7.4 with 0.1 M NaCl (PBS). A control zone was formed using a protein G solution (1 mg/mL, application volume 1 μL/mm) in the same PBS.

GNP conjugates with GAMI (OD_520_ = 2.6, application volume 13 μL/mm) were applied to the conjugate support. After applying the reagents, the membranes were dried in the air at 20–22 °C for at least 20 h. A multimembrane composite was assembled and cut into strips 3.5 mm wide using an Index Cutter-1 automatic guillotine cutter (A-Point Technologies, Gibbstown, NJ, USA).

### 2.7. Honey Samples Preparation

A panel of three honey samples (white honey, floral honey, and acacia honey) for immunochromatography was formed from artificially contaminated honey by diluting them with PBST in a 1:4 ratio (250 mg of honey per 1 mL of 50 mM phosphate buffer, pH 7.4, containing 0.25% Tween-20 [PBST]).

### 2.8. Immunochromatographic Detection of Neomycin

The assay was carried out at room temperature. Anti-NEO antibodies (with a concentration of 18 μg/mL, 25 μL), the NEO–biotin conjugate (concentration 0.3 μM, 25 μL), and PBST or honey samples diluted four times with PBST (50 μL, with NEO in concentrations from 0 to 5 µg/mL) were added to microplate wells and mixed. Test strips were introduced into the wells in a vertical position. After 10 min, the strips were removed and placed on a horizontal surface.

Immunochromatographic detection of streptomycin was carried out as mentioned above. The concentrations of streptomycin added to microplate wells varied from 250 to 15.6 ng/mL; anti-streptomycin antibodies and the streptomycin–biotin conjugate were dropped into the wells in concentrations of 7.5 µg/mL and 10 µg/mL, respectively.

### 2.9. Processing of Test Strips Images and Calculation of the Assay Parameters

After the analysis, the test strips were scanned on a Canon Lide 90 flatbed scanner with a resolution of 600 dpi, without using contrast and color correction modes, and analyzed in the Total Lab software (Nonlinear Dynamics, Newcastle, UK). The total intensities of the line coloration were presented as relative units (RUs), being the same for all data in the paper.

The dependence of line coloration (y) on the antigen concentration in the sample (x) was approximated by the Origin software (OriginLab, Northampton, MA, USA) using a 4-parameter sigmoid function:

y = (a − b)/[1 + (x/c)^d^] + b,

where a = maximal signal, b = minimal signal, c (or IC50) = the antigen concentration that inhibits 50% of antibody binding, and d = the slope of the fitting curve at point c.

Concentrations of the antigen, corresponding to the disappearance of coloration in the analytical zone, were taken as the visual limits of detection. The instrumental limits of detection were calculated from concentration dependences of immunochromatography using 3σ criteria for the difference of the registered coloration from its value for the sample without the analyte [24].

## 3. Results and Discussion

### 3.1. Assay Principle

Unlike the traditional competitive immunochromatography described for a number of antibiotics [25,26,27], including neomycin [28], the proposed test system uses a modular kit of reagents, which makes it possible to combine a universal test strip and specific reagents without their immobilization (Figure 1A). During test strip preparation, streptavidin and streptococcal protein G were immobilized in the analytical and control zones, respectively, and a conjugate of GNPs with anti-species antibodies was applied to the conjugate support. Anti-NEO antibodies and a hapten–biotin conjugate were used as a pair of specifically interacting immunoreagents. Mixing their solutions with the NEO-containing sample initiated immune interactions. Then, after placing the test strip into the reaction mixture and moving this mixture along the test strip driven by capillary forces, the formed immune complexes reacted with streptavidin in the analytical zone and were visualized by the conjugate of GNPs with anti-species antibodies. Note that in the given assay format, the use of universal reagents (biotin, streptavidin, anti-species antibodies, and protein G) makes it possible to quickly switch from one detectable compound to another, replacing only the antibody/biotinylated antigen pair added to the reaction medium.

When a sample is added in the absence of an analyte (Figure 1B), it is mixed with the hapten–biotin conjugate with specific antibodies, and upon further washing out of the GNP conjugate with anti-species antibodies, a triple complex of hapten–biotin–anti-hapten-GNP-anti-species antibodies is formed, which, upon reaching the analytical zone, leads to its staining due to binding with streptavidin.

When the concentration of the analyte in the sample exceeds the threshold value (Figure 1C), the molecules of the analyte block the antibody binding sites, and the formed complex of analyte-specific antibodies further interacts with the GNP-anti-species antibody conjugate. In the zone with the immobilized protein G, the complex with the labeled conjugate is formed.

### 3.2. Choice of Reagents Concentrations for the Universal Test Strip

**Figure 2:**
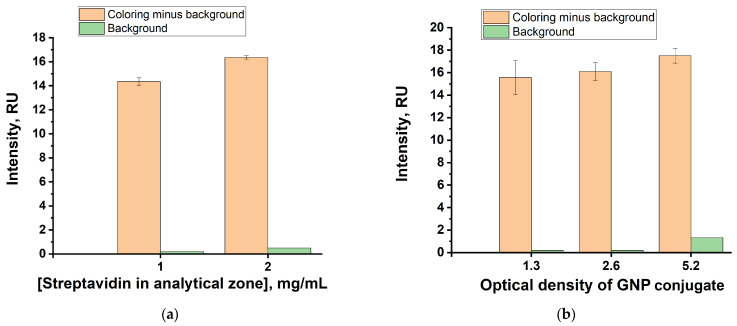
Intensities of the analytical zone coloration for varied concentrations of streptavidin (**a**) and GNP conjugate with anti-species antibodies (**b**).

The concentrations of the GNPs conjugate with anti-species antibodies and streptavidin applied to the analytical zone were optimized. The concentrations of reactants used in the preparation of the universal test strip were varied to reach conditions for efficient detection of the assay results. For this, two concentrations of streptavidin were applied to the analytical zone (1 and 2 mg/mL) and three dilutions of the GNPs conjugate with anti-species antibodies (OD_520_ = 1.3; 2.6 and 5.2) were tested. The coloration of the analytical zone and background were registered for these variants and is presented in Figure 2.

The data obtained for the given concentrations demonstrate key changes influencing the specific and non-specific coloration and reasons to choose optimal values. Thus, in the case of streptavidin (Figure 2a), lowering its concentration from 2 to 1 mg/mL reduced background coloration to an almost unobservable level that did not affect the registration of the specific binding. At the same time, specific coloration for the compared variants decreased by 15%. Since at lower concentrations of streptavidin, the detection of the formed specific complexes will worsen without affecting the background, the concentration of 1 mg/mL was chosen as optimal. A comparison of three variants differing in the concentration of the GNP conjugate used (Figure 2b) allows us to draw the following conclusions: The transition from the variant with OD_520_ = 2.6 to the variant with OD_520_ = 5.2 causes a sharp increase in the background and is therefore unacceptable. On the transition from OD_520_ = 2.6 to OD_520_ = 1.3, the decrease in the specific signal is statistically unreliable. However, its variability increases greatly, which visually corresponds to a less uniform coloration of the analytical zone. Therefore, we settled on the optimal concentration of the conjugate corresponding to OD_520_ = 2.6.

### 3.3. Choice of Concentrations of Neomycin-Specific Reagents

**Figure 3:**
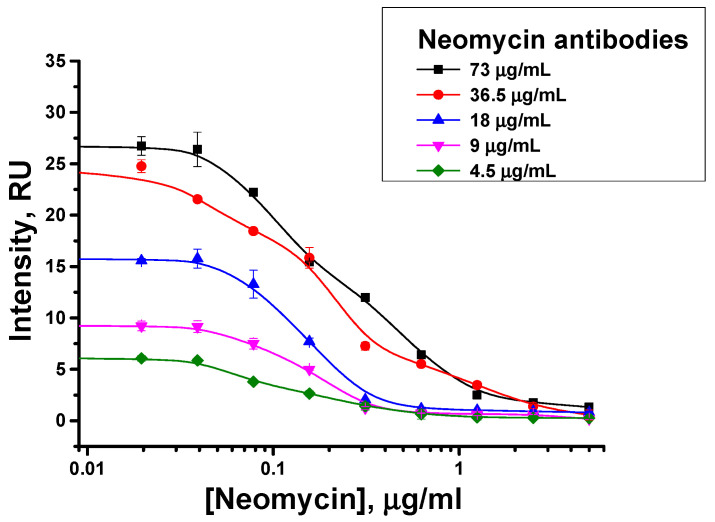
Varying concentration of specific antibodies in the proposed immunochromatographic assay of neomycin: Dependences of coloration on the neomycin concentration (concentration of hapten–biotin conjugate is 0.3 µM).

The dependence of the test system characteristics on the concentrations of specific antibodies and the hapten–biotin conjugate was studied. With a decrease in the concentration of specific antibodies, the color intensity of the analytical zone decreases, but the inflection point of the concentration dependence (IC50) shifts toward lower analyte concentrations (Figure 3). Quantitative parameters of the obtained concentration dependence are integrated into Table 1. As can be seen, with a decrease in the concentration of specific antibodies from 73 to 4.5 µg/mL, there is a decrease in the visual detection limit from 2.5 to 0.3 µg/mL and in the instrumental detection limit from 100 to 60 ng/mL. However, lower concentrations of the antibodies lead to significantly less intense coloration in the absence of the analyte, which makes the visual assessment of the assay results more difficult. Therefore, the concentration of specific antibodies (18 μg/mL) was established, which ensures good sensitivity and acceptable coloration in the analytical zone.

**Figure 4:**
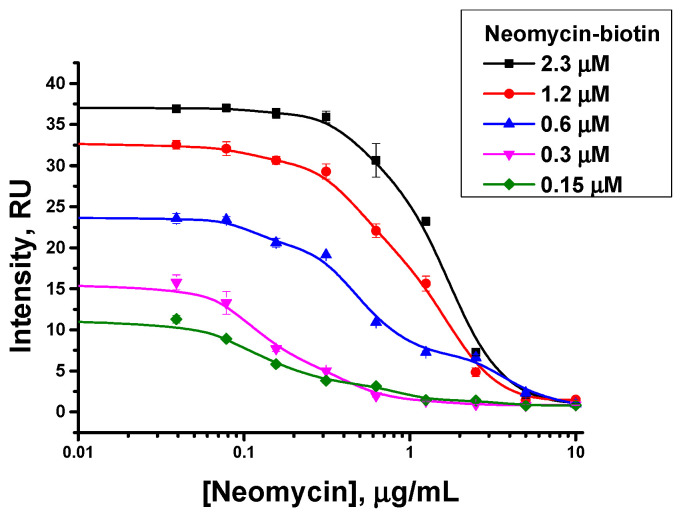
Varying concentration of the hapten–biotin conjugate in the proposed immunochromatographic assay of neomycin: Dependencies of coloration on the neomycin concentration (concentration of specific antibodies is 18 µg/mL).

A decrease in the concentration of the hapten–biotin conjugate also led to a decrease in the visual limit of detection, but decreased coloration of the analytical zone makes it difficult to interpret the analysis results (Figure 4). A conjugate concentration equal to 0.3 µM was chosen, which provided a reliable determination of the target analyte at low concentrations (Table 1).

The presented dependencies demonstrate a common trend described in earlier studies of competitive immunochromatography [29,30,31]. An increase in the concentration of an antigen derivative that competes with the antigen in the sample makes it possible to form a larger number of labeled immune complexes, i.e., leads to a higher amplitude of concentration dependences. However, at a high concentration of the competitor, a higher concentration of antigen in the sample is needed to cause a reliably observable decrease in the registered number of labeled immune complexes (intensity of coloration). Therefore, for experiments with a high concentration of the antigen conjugate, the limit of detection of free antigen in the sample becomes higher [29].

**Figure 5:**
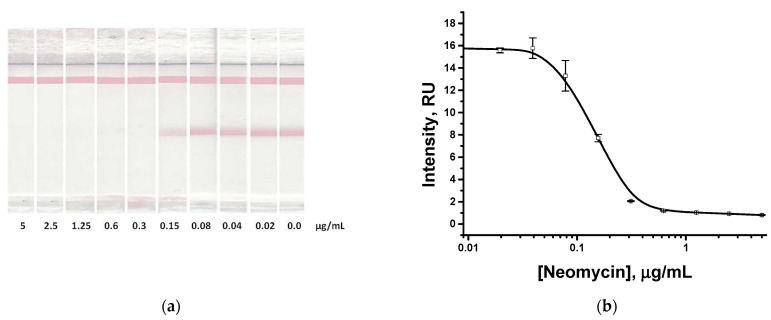
Immunochromatographic detection of neomycin in PBST. Test strips after analysis of samples with different neomycin concentrations (**a**); and the dependence of the analytical zone coloration on the neomycin concentration (**b**). All experiments were carried out in triplicate.

Under the chosen conditions, an immunochromatographic determination of neomycin in the solutions containing an analyte at concentrations from 5 to 0.02 µg/mL was performed. As follows from the obtained results (Figure 5), the visual and instrumental detection limits reached were 0.3 and 0.07 µg/mL, respectively.

### 3.4. Approbation of the Proposed Assay for Honey Testing

**Figure 6:**
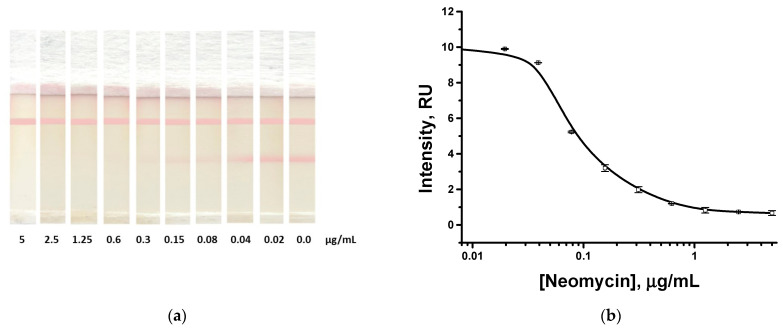
Immunochromatographic detection of neomycin in honey. Test strips after analysis of samples with different neomycin concentrations (**a**); and the dependence of the analytical zone coloration from the neomycin concentration (**b**). All experiments were carried out in triplicate.

The developed assay principle should be integrated with the protocol for real sample testing to exclude risks of significant sample matrix influence on the assay results. The corresponding approbation of the test system was carried out using artificially contaminated samples of honey (containing neomycin at concentrations from 5 to 0.02 µg/mL), previously diluted with PBST in a ratio of 1:4. The visual limit of detection according to the disappearance of coloration in the analytical zone was 0.3 µg/mL (0.3 mg/kg; Figure 6a), and the instrumental detection limit was 0.014 µg/mL (0.014 mg/kg; Figure 6b). Thus, the detection limits reached are in the accordance with regulatory demands concerning permissible levels of neomycin in food [32]. The presence of honey compounds in the tested solution increases its viscosity. As a result, the flow of reagents along the working membrane slows down, which was observed in our experiments. As a result, the reaction proceeds under conditions closer to equilibrium. These reasons for the more efficient binding of immune reactants during their lateral flow can cause a shift in the detection limit towards lower concentrations.

The characterization of spiked honey samples (three tested concentrations with three repetitions) demonstrated that the revealing of neomycin in honey was in the range of 85–113% (Table 2). This fact demonstrates that the simple pretreatment of honey samples used limited by its 5-fold dilution is sufficient for reliable control and making decisions regarding levels of contamination.

### 3.5. Demonstration of the Universality of the Proposed Assay for Streptomycin Detection

**Figure 7:**
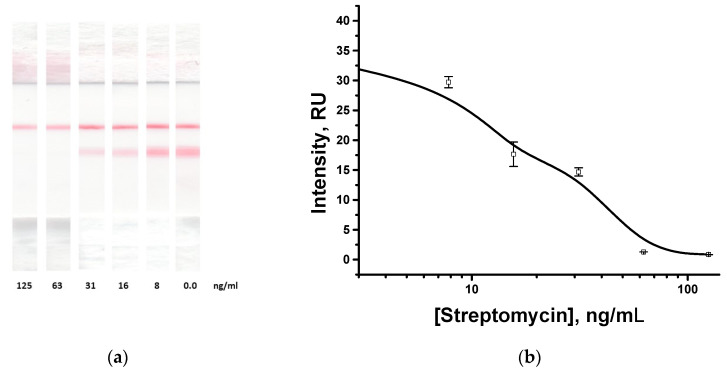
Immunochromatographic assay of streptomycin using the proposed modular approach. Test strips after analysis of samples with different streptomycin concentrations (**a**); and the dependence of the analytical zone coloration on the streptomycin concentration (**b**). All experiments were carried out in triplicate.

To confirm the universality of the proposed approach, immunochromatographic detection of the antibiotic streptomycin was carried out. The visual limit of detection was 63 ng/mL (Figure 7). The results obtained prove that this format is suitable for different analytes, and the chosen completion of the universal test strip allows efficient work with new analyte-specific reactants—intense coloration without the analyte and complete disappearance of the coloration for high analyte concentrations. The visual limit of detection according to the disappearance of coloration in the analytical zone was 63 ng/mL (Figure 7a), and the instrumental detection limit was 7 ng/mL (Figure 7b). The lower visual limit of detection for the streptomycin case as compared with neomycin could be interpreted as a result of different affinities for two pairs of immune reactants.

## 4. Conclusions

A scheme of modular competitive immunochromatography, based on the use of universal reactants such as streptavidin-biotin and anti-species antibodies is presented. Streptavidin–biotin complexes provide one of the strongest interactions and are commonly used in immunoassays [33]. The presented approach is tested on the example of the detection of neomycin in honey. The visual and instrumental detection limits were 0.3 and 0.014 mg/kg, respectively, in accordance with the regulatory demands for food control. The proposed modular competitive immunochromatography separates analyte-specific reactants that are mixed in the diluting solution and a universal test strip containing only analyte-independent reactants. This decision makes it possible to overcome the disadvantages of traditional competitive immunochromatography related to the necessity of an additional choice of conditions for the immobilization of specific immune reactants with the risk of their inactivation. In this way, the assay transfer to the other analyte is reduced to choosing two concentrations: For specific antibodies and the hapten–biotin conjugate.

## Figures and Tables

**Table 1:** Visual and instrumental detection limits for immunochromatographic detection of neomycin under varied conditions.

Anti-Neomycin Antibodies, μg/mL	Neomycin-Biotin, µM	Visual Detection Limit, ng/mL	Instrumental Detection Limit, ng/mL
Experiments with varied anti-neomycin antibodies concentration (Figure 4)			
73	0.3	2.5	0.1
36.5	0.3	2.5	0.06
18	0.3	0.3	0.07
9	0.3	0.3	0.07
4.5	0.3	0.3	0.06
Experiments with varied neomycin-biotin concentration (Figure 5)			
18	2.3	5	0.67
18	1.2	5	0.49
18	0.6	5	0.32
18	0.3	0.3	0.07
18	0.15	0.3	0.06

**Table 2:** Recoveries of neomycin in honey.

Neomycin Added, ng/mL	Neomycin Detected, ng/mL	Recovery, %
19	21	110
39	33	85
78	88	113

## Data Availability

The data that support the findings of this study are available from the corresponding author upon request.

## Supplementary Materials

The following supporting information can be downloaded at: https://www.mdpi.com/article/10.3390/bios13050498/s1, Figure S1: Micrograph of GNP obtained by transmission electron microscopy (a) and histogram of diameter distribution of nanoparticles (b). Reference [34] is cited in the Supplementary Materials.
