# Supplementary material for: Modular Set of Reagents in Lateral Flow Immunoassay: Application for Antibiotic Neomycin Detection in Honey
Source: Biosensors (Basel). 2023 Apr 25;13(5):498. doi: 10.3390/bios13050498 (PMC10216841; doi:10.3390/bios13050498)
Supplement: Supplementary file 1 [file biosensors-13-00498-s001.zip › biosensors-2325246-Supplementary -english done.pdf]

biosensors

Supplementary Materials

Modular Set of Reagents in Lateral Flow Immunoassay: Application for Antibiotic Neomycin Detection in Honey

Dmitriy V. Sotnikov[1], Lyubov V. Barshevskaya[1], Anatoly V. Zherdev[1], Boris B. Dzantiev[1*]

1 A.N. Bach Institute of Biochemistry, Research Center of Biotechnology of the Russian Academy of Sciences, Leninsky prospect 33, Moscow 119071, Russia
* Correspondence: dzantiev@inbi.ras.ru, Tel.: +7-495-954-31-42

Obtainment and characterization of the gold nanoparticles

GNPs were synthesized by the Frens method [1], which makes it possible to obtain particles of a certain size depending on the amount of the added reducing agent (citrate salt). The ratio of reactants was chosen on the basis of our earlier application of the Frens method [2] and the known recommendations that the optimal diameters of GNPs for immunochromatography are in the range of 20–40 nm [3, 4].

The obtained preparation of GNPs was characterized by transmission electron microscopy. According to the obtained data (Figure S1), the average diameter of the nanoparticles was 18.3 ± 2.3 nm (n = 120; the minimum value was 10.1 nm and the maximum value was 27.8 nm), with a degree of ellipticity of 1.12 ± 0.06.

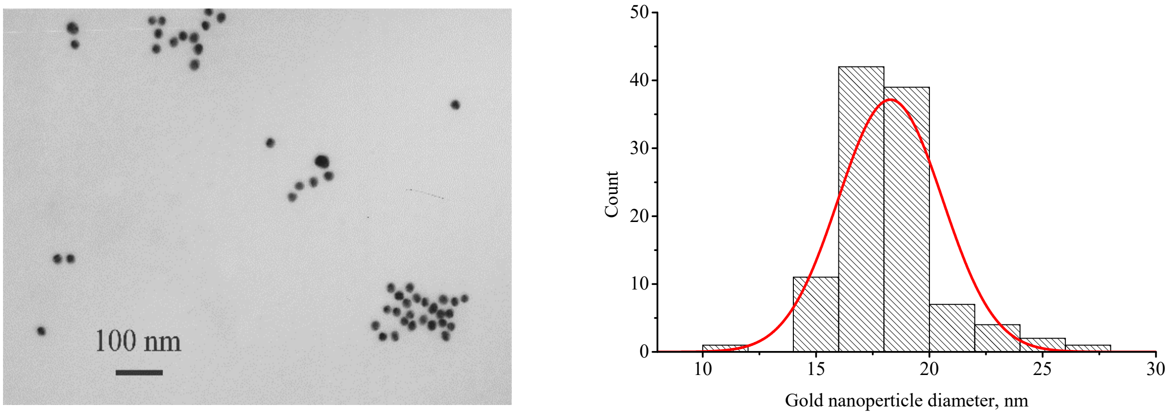

(**a**) (**b**)

Figure S1. Micrograph of GNPs obtained by transmission electron microscopy (a) and histogram of diameter distribution of nanoparticles (b).

References

1. Frens, G., Controlled nucleation for the regulation of the particle size in monodisperse gold suspensions. *Nat. Phys.*, **1973,** *241(105)*, 20-22. https://doi.org/10.1038/physci241020a0
2. Safenkova, I.V., Zherdev, A.V., Dzantiev, B.B., Factors influencing the detection limit of the lateral-flow sandwich immunoassay: a case study with potato virus X. *Anal Bioanal Chem.*, **2012,** *403(6)*, 1595-1605. https://doi: 10.1007/s00216-012-5985-8
3. Parolo, C., Sena-Torralba, A., Bergua, J.F., Calucho, E., Fuentes-Chust, C., Hu, L., Rivas, L., Álvarez-Diduk, R., Nguyen, E.P., Cinti, S., Quesada-González, D., Merkoçi, A., Tutorial: design and fabrication of nanoparticle-based lateral-flow immunoassays. *Nat Protoc.*, **2020,** *15(12)*, 3788-3816. https://doi: 10.1038/s41596-020-0357-x
4. Wang, Z., Zhao, J., Xu, X., Guo, L., Xu, L., Sun, M., Hu, S., Kuang, H., Xu, C., Li, A., An overview for the nanoparticles-based quantitative lateral flow assay. *Small Methods*, **2022,** *6(1)*, 2101143. https://doi: 10.1002/smtd.202101143
